# Traditional Chinese medicine on treating diabetic mellitus erectile dysfunction: Protocol for a systematic review and meta-analysis

**DOI:** 10.1097/MD.0000000000014928

**Published:** 2019-03-15

**Authors:** Xudong D. Yu, Jisheng S. Wang, Guang Zuo, Xin Wang, Fuxing Ge, Songli Wu, Jingyang Lim, Jianwei Shang, Yaosheng Zhang

**Affiliations:** a Graduate School of Beijing University of Chinese Medicine; b Department of Andrology, Dongzhimen Hospital, Beijing University of Chinese Medicine, Beijing; c Department of Orthopedics, Hebei Traditional Chinese Medicine Hospital, Hebei University of Traditional Chinese Medicine, Shijiazhuang City, Hebei; d Department of Periangiaceae, Dongzhimen Hospital, Beijing, China.

**Keywords:** diabetic mellitus erectile dysfunction, protocol, systematic review, traditional Chinese medicine

## Abstract

**Background:**

Diabetic mellitus erectile dysfunction (DMED) refers to erectile dysfunction (ED) secondary to diabetes. As people's lifestyle changes and the population ages, the incidence of DMED continues to increase. Many clinical trials have proven that Chinese medicine has a significant effect in the treatment of DMED. In this systematic review, we aim to evaluate the effectiveness and safety of traditional Chinese medicine (TCM) for DMED.

**Methods:**

We will search PubMed, Cochrane Library, AMED, EMbase, WorldSciNet; Nature, Science online and China Journal Full-text Database, China Biomedical Literature CD-ROM Database, and related randomized controlled trials included in the China Resources Database. The time is limited from the construction of the library to February, 2019.We will use the criteria provided by Cochrane 5.1.0 for quality assessment and risk assessment of the included studies, and use the Revman 5.3 and Stata13.0 software for meta-analysis of the effectiveness, recurrence rate, and symptom scores of DMED.

**Ethics and dissemination:**

This systematic review will evaluate the efficacy and safety of TCM for treating Diabetic mellitus erectile dysfunction. Because all of the data used in this systematic review and meta-analysis has been published, this review does not require ethical approval. Furthermore, all data will be analyzed anonymously during the review process trial.

## 1 Introduction

Diabetic mellitus erectile dysfunction (DMED) refers to erectile dysfunction (ED) secondary to diabetes. It is characterized by persistent or repetitive penile erection and insufficient hardness or sufficient time to be satisfied. The phenomenon of completing sexual activity.^[1,2]^ It is a type of diabetic sexual dysfunction.^[3]^ With the improvement of people's living standards, the incidence of diabetes, especially type 2 diabetes, has been increased, and the complications it has brought cover multiple organs of the human body.^[4,5]^ ED is 1 of its common complications. Surveys in the United Kingdom, the United States, and other countries have shown that the incidence of erectile dysfunction in the normal population is 0.1% to 18%. However, the incidence of ED in diabetic patients has increased nearly 3-fold compared with the normal population, and tends to be in younger population.^[6]^ Studies have shown that the number of people with ED in diabetes has reached 71% in the past 10 years. Diabetes ED patients often have severe symptoms and are a type of refractory ED, which seriously affects the quality of lives of the diabetic patients.^[7]^

The pathogenesis of DMED is not fully understood. At present, most scholars believe that high glucose environment leading to central nervous system and pericardial nerve damage is an important cause of the disease.^[8]^ Some scholars believe that diabetes causes penile artery blood supply and venous closure to cause ED.^[9]^ Apart from this, age, duration of diabetes, control of blood glucose, smoking, hypertension, atherosclerosis, adverse drug reactions, and psychological factors are all closely related to the development of DMED. DMED not only seriously deteriorates the quality of life of patients, the harmony between family and society, but also causes greater physical and mental pain to male patients.^[10]^ The rapid development of material civilization and spiritual civilization is increasingly concerned by the society for the prevention and treatment of DMED. The current treatment methods for DMED can be roughly summarized as primary disease treatment, psychotherapy, and symptomatic treatment. These include basic treatment (eliminating risk factors such as controlling blood sugar, etc), psychotherapy, and medication (PDE-5 inhibitors, etc).^[11,12]^ However, due to the complexity of the pathogenesis of DMED, there is still no specific treatment for DMED in modern medicine. Studies have shown that PDE-5 inhibitors have side effects such as headache, facial redness, and gastrointestinal reactions during the treatment of DMED.^[13]^ Apart from these, in recent years, the studies have reported that some patients have prolonged drug onset time and drug resistance during the medication.^[14]^ Diabetes belongs to the category of “Xiaoke” in traditional Chinese medicine (TCM). Male erectile dysfunction belongs to the category of “yangshuo” in TCM. TCM believes that DMED lesions are mainly in the kidney, “yin deficiency with internal heat,” and “qi stagnation and blood stasis” is its main pathogenic factor.^[15,16]^ With the application of TCM in the treatment of DMED, a unique diagnosis and treatment system, clinical efficacy is significant. Modern research shows that the effective active ingredients in Chinese medicine can not only promote the activity of sputum-glucosidase, but also improve the blood supply of peripheral blood vessels, so as to achieve the purpose of treatment.^[17,18]^ TCM regulates body function as a whole through multifaceted and multitarget mechanism, and has unique advantages in the treatment of DMED.^[19]^

After a preliminary search and analysis of database resources, we found that randomized controlled trials (RCTs) of TCM for DMED are gradually increasing. However, due to the size and number in clinical trials, most clinical trials face problems with small sample size and evidence-based exploration. Therefore, we expect a meta-analysis of relevant studies on TCM treatment of DMED and a meta-analysis to assess its clinical efficacy and safety.

## 2 Methods

This is a systematic review and ethical approval was not necessary.

### 2.1 Study registration

This systematic review protocol has been registered on PROSPERO as CRD42019117444 (hops://www.crd.york.cord.php?RecordlD=17444).

### 2.2 Eligibility criteria

#### 2.2.1 Type of study

Take TCM or Chinese medicine combined with other effective interventions as main treatment, including RCTs of the control group (effective methods other than TCM). Language is limited in Chinese and English. Non-RCTs, quasi-RCTs, case series, case reports, and crossover studies will be excluded.

#### 2.2.2 Participants

Men with a history of diabetes who match the Diagnostic Criteria for Diabetes: refer to the American Diabetes Association Diabetes Care Guidelines. The diagnosis is ED after diabetes, and the International Index of Erectile Function 5 (IIEF-5) score is <21. The course of ED is ≥3 months. The patient must be at least 18 years of age. The sexual partners of the patients are fixed. The group is well balanced when enrolled.

#### 2.2.3 Types of interventions

##### 2.2.3.1 Experimental interventions

The drug composition, the dose-specific Chinese medicine preparation or the combined western medicine, is used as experimental interventions. Both prescription and Chinese patent medicines will be included. Other TCM treatments such as intravenous medication, acupuncture, and massage will be limited.

##### 2.2.3.2 Control interventions

As for the control interventions, those who accepted simple western medicine can be used as a control intervention or those who did not get any treatment as a blank control would be adopted. However, once they had accepted the therapy of TCM, the trials will be rejected.

#### 2.2.4 Outcomes

The primary outcome measurement will be assessed using the International Erectile Function Index.

Healing: IIEF-5 score ≥22 points after treatment; significant effect: IIEF-5 score <22 points after treatment, score improvement ≥60%; effective: IIEF-5 score <22 points after treatment, points improved <60%, but ≥30%; invalid: IIEF-5 score <22 points after treatment, score improvement <30%.

The secondary outcome measurement will be assessed according to the TCM syndrome scoring criteria.

Healing: The clinical symptoms and signs of TCM disappear or disappear, and the syndrome score is reduced by ≥90%; markedly effective: the clinical symptoms and signs of TCM are obviously improved, the syndrome score is reduced by ≥60%; effective: TCM clinical symptoms signs and signs have improved, syndrome points reduced by <60%, but ≥30%; invalid: Chinese clinical symptoms and signs have not improved, or even worse, syndrome scores reduced by <30%. Integral variation formula (Nimodipine method [{pretreatment score − post-treatment score} ÷ pretreatment score] × 100%).

#### 2.2.5 Data source

##### 2.2.5.1 Electronic searches

The electronic search database includes PubMed, Cochrane Library, AMED, EMbase, WorldSciNet, Nature, Science online and China Journal Full-text Database, China Biomedical Studies CD-ROM Database (CBM), and China Resources Database. The clinical research studies on the treatment of Diabetic mellitus erectile dysfunction with Chinese medicine published in domestic and foreign biomedical journals from the establishment of the library to February, 2019 was searched. Based on the standards of the Cochrane Collaboration Workbook of the International Evidence-Based Medicine Center, a manual and computer-based method is used to conduct related studies. The search terms include: Chinese medicine, traditional Chinese medicine, proprietary Chinese medicine, Chinese herbal medicine, Diabetic mellitus erectile dysfunction, impotence. According to the characteristics of different databases, comprehensive search using keyword was conducted. All search results are determined after multiple searches. We will follow the references included in the studies to incorporate relevant studies as much as possible to avoid missed detection. The search term in the Chinese database is the translation of the above word. The complete PubMed search strategy is summarized in Table 1.

**Table 1:**
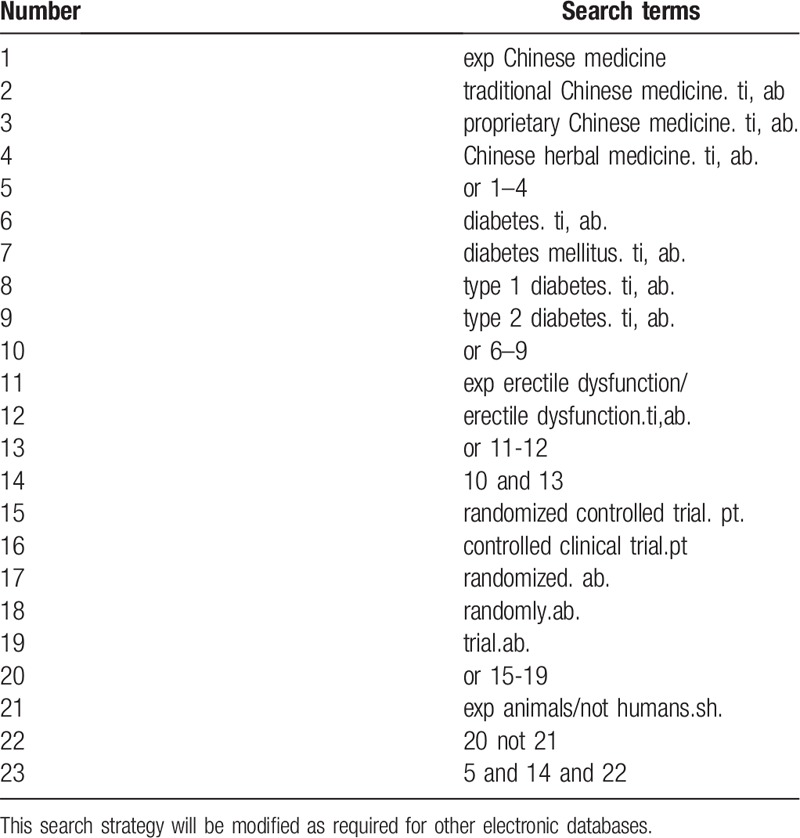
Search strategy used in PubMed database.

##### 2.2.5.2 Searching other resources

The manual search mainly is used for searching relevant studies such as “Chinese Journal of Endocrinology and Metabolism,” “National Journal of Andrology,” and “Chinese Journal of Urology” before the database creation.

#### 2.2.6 Data collection and analysis

**Figure 1:**
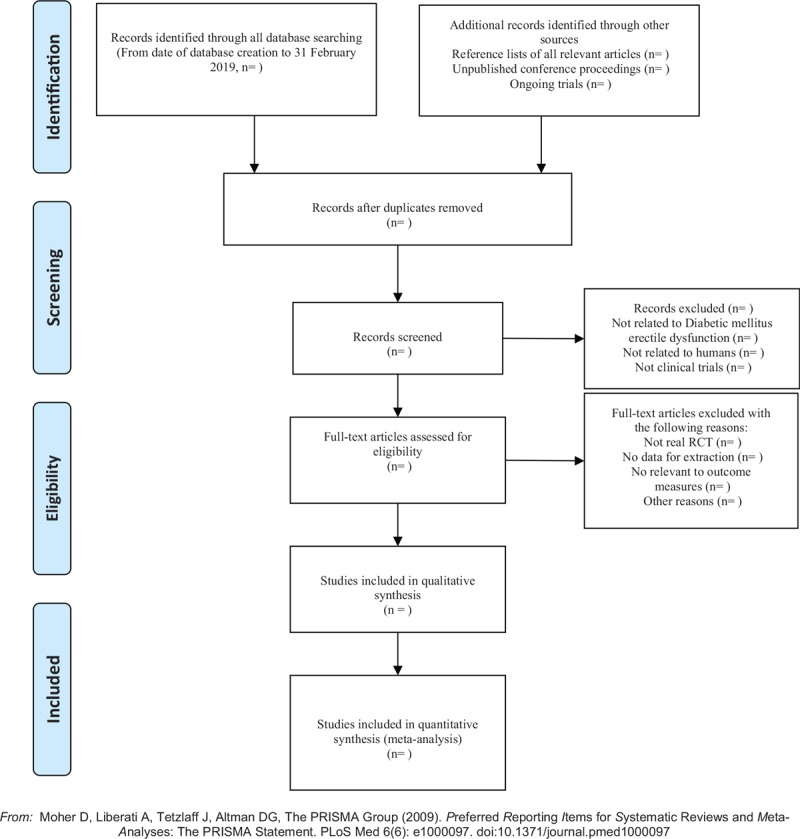
The PRISMA flow chart.

The data collection and analysis was conducted as follows:

1. Applying the EndnoteX7 software to manage the included references. Two qualified evaluators independently screened the titles and abstracts of the selected studies, excluding duplicates and documents that did not significantly conform to the study.
2. The second screening of the studies: Screening out unqualified studies such as case report, theoretical discussion, and nonconformance of interventions was done. After preliminary evaluation, the remaining studies to further screen out the unqualified studies such as ordinary control study, no control group, no random grouping, no outcome index, and data mine equivalent were carefully read.
3. For the studies that could not be determined whether to be included or not, it was decided by 2 researchers. If the opinions were not uniform, third-party expert was asked. A clinical RCT was finally included in the study.
4. Information and data extraction for the final included documents: The extracted data and information included the test methods of the study, the basic information of the included cases, the observation period, the intervention methods of the treatment group and the control group, the observation indicators, and the test results (Fig. 1).

#### 2.2.7 Risk of bias

The studies quality assessment applies the bias risk assessment tool recommended by Cochrane to evaluate the quality of all included studies and the risk of bias. The assessment will include sequence generation, allocation concealment, blinding of participants, personnel and outcome assessors, incomplete outcome data, selective outcome reporting, and other sources of bias. The risk of high and low bias is expressed as “high risk” and “low risk,” respectively. The information provided in the study is inaccurate or does not provide sufficient information for the bias assessment to be expressed as “unclear risk.” The above content evaluation is independently evaluated by 2 researchers. If there are different opinions, the discussion will be conducted. If there are still differences, a third appraiser will be consulted. Otherwise, the Cochrane Professional Group will be consulted.

#### 2.2.8 Statistical analysis

The meta-analysis in this study will use Rev Man5.3 and Stata13.0 statistical software. Heterogeneity tests will be used for the included experimental studies. The numerical variable will be expressed as the normalized mean difference with a confidence interval (CI) of 95%. The heterogeneity of each pair-wise comparison will be tested by chi-square test (test level α = 0.1). If there is no heterogeneity, a fixed-effect model will be used. If there is significant heterogeneity between a set of studies, we will use a random-effects model (REM) for meta-analysis. We will explore the reasons for the existence of heterogeneity from various aspects such as the characteristics of the subjects and the degree of variation of the interventions. The source of heterogeneity is further determined by means of sensitivity analysis.

#### 2.2.9 Publication bias

If a result of a meta-analysis contains more than 10 articles and above, we will use a funnel plot to test the risk of publication bias. Quantitative methods such as Begg testing and Egger testing will be used to help assess publication bias in the application.

##### 2.2.9.1 Quality of evidence

The Grading of Recommendations Assessment, Development and Evaluation (GRADE) method will be used to assess the quality of evidence for key outcomes. This assessment will be conducted through a Guideline Development Tool (GRADEpro GDT, https://gradepro.org/).

## 3 Discussion

In recent years, with the changes in people's lifestyles and the aging of the population, the incidence of diabetes has increased. Diabetes has a variety of chronic complications, including heart disease, high blood pressure, stroke, and ED. Among them, diabetes is most closely related to ED and is the leading cause of organic ED.^[20,21]^ About half of the diabetic patients have ED. TCM believes that the etiology and pathogenesis of ED is related to spleen and kidney deficiency, and qi and blood block. TCM can play the role of strengthening the spleen and kidney, promoting blood circulation and collaterals, and at the same time, can improve the mood and achieve the purpose of treatment.^[22–24]^ Modern research shows that the effective active ingredients in Chinese medicine can not only promote the activity of sputum-glucosidase, but also improve the blood supply of peripheral blood vessels, so as to play a corresponding therapeutic role.^[25,26]^

With the deepening of understanding of diabetes and its complications, the trials and clinical reports of TCM treatment of DMED have gradually increased. Whether it is syndrome differentiation or special disease, Chinese medicine has achieved good results in the treatment of diabetes ED. To the best of our knowledge, there has been no comparison of the efficacy and safety of TCM for the treatment of DMED in recent years. Therefore, we will compare the effectiveness and safety of TCM in the treatment of DMED by applying systematic evaluation and meta-analysis. The results of this study can provide a possible ranking for Chinese medicine treatment of DMED. We hope that the results will provide clinicians with the best options for treating DMED and provide them with research directions. Although we will conduct a comprehensive search in this study, languages other than Chinese and English will be restricted, which will lead to some bias. In addition, the relevant studies on TCM treatment of DMED are small and the overall quality is low, which may affect the authenticity of this study. Therefore, we hope that in the future, we will have a more rigorous and reasonable multicenter RCT to explore the clinical efficacy of TCM in the treatment of DMED, so that the conclusion is more objective and reasonable.

## Author contributions

**Conceptualization:** Jianwei Shang.

**Formal analysis:** Xudong D. Yu, Jisheng S. Wang, Songli Wu.

**Funding acquisition:** Xudong D. Yu, Jisheng S. Wang, Jianwei Shang.

**Investigation:** Jisheng S. Wang.

**Project administration:** Jisheng S. Wang.

**Supervision:** Xudong D. Yu, Jisheng S. Wang, Yaosheng Zhang.

**Validation:** Xudong D. Yu, Jisheng S. Wang, Jingyang Lim.

**Writing – original draft:** Xudong D. Yu, Jisheng S. Wang, Guang Zuo, Fuxing Ge.

**Writing – review & editing:** Xudong D. Yu, Jisheng S. Wang, Xin Wang, Fuxing Ge.
